# Potential Natural Blend Hydrosol TGLON Suppresses the Proliferation of Five Cancer Cell Lines and Also Ameliorates Idiopathic Pulmonary Fibrosis in a Mouse Model

**DOI:** 10.3390/ph18060872

**Published:** 2025-06-11

**Authors:** Wei-Hsiang Huang, Mei-Lin Chang, Ching-Che Lin, Chih-Peng Wang, Feng-Jie Tsai, Chih-Chien Lin

**Affiliations:** 1 Department of Cosmetic Science, Providence University, No. 200, Section 7, Taiwan Boulevard, Shalu District, Taichung City 433303, Taiwan; weihsiang84@pu.edu.tw (W.-H.H.); tftsay@pu.edu.tw (F.-J.T.); 2 Guidance Association of Taiwan Aromaplants (GATA), R1F., No. 132-9, Tianmu W. Rd., Beitou District, Taipei City 112063, Taiwan; office@gata.org.tw; 3 Holy Tree Biomedical Co., Ltd., No. 132-9, Tianmu W. Rd., Beitou District, Taipei City 112063, Taiwan; 4 Feeoils Aromatherapy Co., Ltd., No. 61-1, Gangtou Ln., Guoxing Township, Nantou County 544005, Taiwan; 5 Department of Chemical Engineering and Biotechnology, National Taipei University of Technology, No. 1, Section 3, Jhongsiao E. Rd., Da’an District, Taipei City 106344, Taiwan; chingche@ntut.edu.tw; 6 Brion Research Institute of Taiwan, 3F., No. 207, Section 3, Beixin Rd., Xindian District, New Taipei City 231033, Taiwan; 7 Institute for Drug Evaluation Platform, Development Center for Biotechnology, No. 101, Lane 169, Kangning St., Xizhi District, New Taipei City 221013, Taiwan; willpengwang@dcb.org.tw

**Keywords:** blend hydrosol, TGLON, cancer cells, reduce pulmonary fibrosis, natural products

## Abstract

**Background:** Cancer and fibrotic diseases represent major global health challenges, underscoring the need for safe, multifunctional natural therapies. Although natural products possess notable anticancer properties, their clinical translation is often hindered by non-selective cytotoxicity toward normal cells. Moreover, their therapeutic potential against chronic conditions such as idiopathic pulmonary fibrosis (IPF) remains insufficiently explored. This study aimed to evaluate the efficacy and safety of a natural hydrosol blend, The Greatest Love of Nature (TGLON), in inhibiting cancer cell proliferation and mitigating IPF. **Methods:** TGLON, composed of 12 steam-distilled plant hydrosols, was chemically characterized by gas chromatography–mass spectrometry (GC-MS). Its cytotoxicity was assessed using the MTT assay against five human cancer cell lines (A-549, HepG2, MCF-7, MKN-45, and MOLT-4) and normal human lung fibroblasts (MRC-5). In vivo safety and therapeutic efficacy were evaluated in Sprague Dawley rats and a bleomycin-induced IPF mouse model, following protocols approved by the Institutional Animal Care and Use Committee (IACUC). **Results:** TGLON maintained >90% viability in MRC-5 cells at an 80-fold dilution and significantly inhibited the proliferation of A-549 (41%), HepG2 (84%), MCF-7 (50%), MKN-45 (38%), and MOLT-4 (52%) cells. No signs of toxicity were observed in rats administered TGLON orally at 50% (*v*/*v*), 10 mL/kg. In mice, TGLON alleviated bleomycin-induced pulmonary inflammation and fibrosis. **Conclusions:** TGLON exhibited selective anticancer and anti-fibrotic activities under non-toxic conditions, supporting its potential as a bioactive agent for early-stage disease prevention and non-clinical health maintenance.

## 1. Introduction

Essential oils are complex volatile substances extracted from plants via distillation or pressing, whereas hydrosols are by-products of the distillation process. This study predominantly focuses on hydrosols.

According to global cancer statistics from 185 countries, the age-standardized incidence rate (ASR) of lung cancer in 2018 was 31.5 per 100,000 in males and 14.6 per 100,000 in females. Liver cancer was more prevalent in males, with an ASR of 15.5 per 100,000, whereas breast cancer remained the most commonly diagnosed malignancy among females, with an ASR of 46.3 per 100,000. Gastric cancer also constituted a substantial portion of the global cancer burden, with incidence rates of 17.2 and 7.5 per 100,000 in males and females, respectively. Cancer often coexists with hematological disorders such as acute lymphoblastic leukemia, leading to abnormal blood environments and hematopoietic dysfunctions, which weaken the immune system and increase the risk of pathogen infections [1]. Chronic lung cancer complications may relate to idiopathic pulmonary fibrosis, characterized by excessive synthesis of fibrous proteins in the *Extracellular Matrix* (ECM) of the alveoli, resulting in pathological accumulations that harden the lungs [2].

Extracts from aromatic plants have been proven to mitigate idiopathic pulmonary fibrosis and exhibit inhibitory activities against cancer cells [3,4]. For example, *Cymbopogon nardus* (CN) inhibits human prostate cancer cells [5]; *Litsea cubeba* (LC) acts against human lung, liver, breast, and oral cancer cells [6,7]; *Chamaecyparis formosensis* (CF) inhibits human liver cancer cells [8]; *Calocedrus formosana* (CAF) is effective against human lung and bladder cancer cells [9,10]; *Cinnamomum camphora* (CC) suppresses human colorectal cancer cells [11]; *Eucalyptus robusta* (ER) inhibits human pancreatic cancer cells [12]; *Cinnamomum zeylanicum* (CZ) acts against human liver and breast cancer cells [13,14]; *Cunninghamia lanceolata* (CL) inhibits human lung and liver cancer cells [15]; *Melaleuca alternifolia* (MA) targets human lung, breast, and prostate cancer cells [16]; *Cinnamomum micranthum* (CM) inhibits murine leukemia cells [17]; *Cryptomeria japonica* (CJ) suppresses human oral and lung cancer cells [18,19]; and *Acacia confusa* (AC) acts against human breast cancer cells [20]. Although these aromatic plants demonstrate anticancer activities, they may also be toxic to normal cells, such as potential damage to normal human lung cells by tea tree oil [21].

Therefore, the ability to inhibit cancer cells without harming normal cells has become a significant issue. Recent years have seen a focus on “compound essential oils” as a research axis, observing their antimicrobial, antifungal, and antiviral activities, as well as their effects in reducing diarrhea in animal studies, highlighting “compounds” as a novel research trend [22,23]. Simultaneously, we are the first research team to use “blend hydrosols” to study cancer cell inhibition, acute oral toxicity in animals, and the mitigation of idiopathic pulmonary fibrosis.

## 2. Results

### 2.1. GC-MS Analysis of TGLON

Please refer to Table 1, which shows a total of 23 compounds that were identified in TGLON. Detailed structural data, including molecular structures, KI, and identification methods, are provided in the Supplementary Materials (Figure S1 and Table S1). The compounds, listed in descending order of concentration, were as follows: Terpinen-4-ol (10.72%), Camphor (8.73%), δ-Cadinene (7.58%), α-Terpineol (6.93%), Safrole (6.87%), 1,8-Cineole (6.08%), cis-Myrtanol (4.22%), (−)-Myrtenol (3.94%), γ-Terpinene (3.17%), α-Muurolene (2.90%), tau-Cadinol (2.82%), α-Cedrene (2.47%), α-Pinene (2.46%), tau-Muurolol (2.46%), α-Cedrol (2.16%), γ-Muurolene (2.15%), D-Limonene (1.93%), α-Terpinene (1.54%), α-Elemol (1.45%), p-Cymene (1.42%), Borneol (1.12%), β-Citronellal (1.11%), and β-Elemene (0.94%).

When categorized by analog type, the total concentrations were as follows: monoterpenols (26.93%), sesquiterpenes (16.04%), oxides (12.95%), monoterpenes (10.52%), sesquiterpenols (8.89%), ketones (8.73%), and aldehydes (1.11%).

### 2.2. Viability of TGLON in MRC-5 Normal Lung Cells

**Figure 1:**
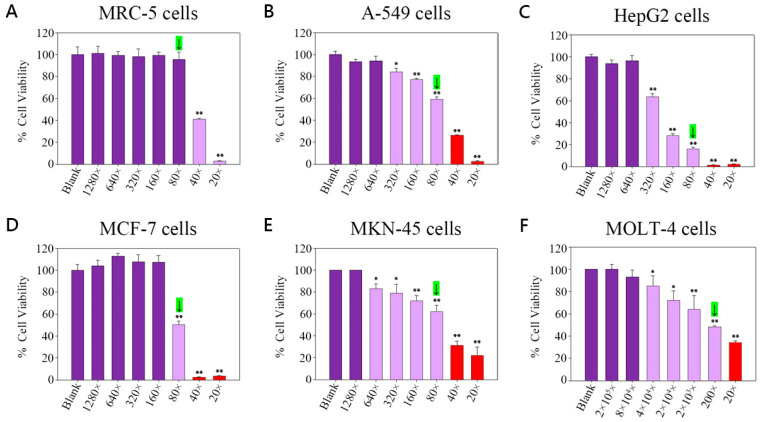
Cell viability of TGLON in (**A**) MRC-5 cells; (**B**) A-549 cells; (**C**) HepG2 cells; (**D**) MCF-7 cells; (**E**) MKN-45 cells; (**F**) MOLT-4 cells. Green bars: final safe dose. Green arrows indicate the final safe concentration. Deep purple bars: unable to inhibit cancer cells. Light purple bars: inhibit cancer cells. Red bars: unsafe for normal cells. Data are presented as means ± SD (*N* = 3). ** *p* < 0.01, * *p* < 0.05 compared to the Blank group. Only statistically significant differences deemed biologically relevant are annotated.

Refer to Figure 1A, which shows that the viability of MRC-5 cells (% mean ± SE) was as follows: Blank (only cells) was 100.00 ± 7.10; at a 1280-fold dilution of TGLON, viability was 101.00 ± 6.70; at 640-fold dilution, it was 99.10 ± 3.90, indicating approximately 1% inhibition; at 320-fold dilution, it was 98.00 ± 7.00, indicating approximately 2% inhibition; at 160-fold dilution, it was 99.20 ± 3.10, indicating approximately 1% inhibition; at 80-fold dilution, it was 95.40 ± 6.90, indicating approximately 5% inhibition; at 40-fold dilution, it was 40.90 ± 1.00, indicating approximately 59% inhibition; and at 20-fold dilution, it was 2.70 ± 0.30, indicating approximately 97% inhibition.

Cell viability of MRC-5 cells remained above 90% in dilutions from 1280× to 80×, with no significant difference from the control group (*p* > 0.05). In contrast, 40× and 20× resulted in significant cytotoxicity (*p* < 0.01). Therefore, 80× was designated as the final safe concentration.

### 2.3. Viability of TGLON in A-549 Lung Cancer Cells

Refer to Figure 1B, where the viability of A-549 cells (% mean ± SD) was as follows: Blank (only cells) was 100.00 ± 2.90; at a 1280-fold dilution of TGLON, viability was 93.20 ± 2.40, indicating approximately 7% inhibition; at 640-fold, it was 94.10 ± 4.40, indicating approximately 6% inhibition; at 320-fold, it was 84.00 ± 3.60, indicating approximately 16% inhibition; at 160-fold, it was 77.00 ± 1.20, indicating approximately 23% inhibition; at 80-fold, it was 59.30 ± 2.20, indicating approximately 41% inhibition; at 40-fold, it was 26.50 ± 0.30, indicating approximately 73% inhibition; and at 20-fold, it was 2.30 ± 0.70, indicating approximately 98% inhibition.

### 2.4. Viability of TGLON in HepG2 Liver Cancer Cells

Refer to Figure 1C, where the viability of HepG2 cells (% mean ± SD) was as follows: Blank (only cells) was 100.00 ± 2.30; at a 1280-fold dilution of TGLON, viability was 93.80 ± 3.20, indicating approximately 6% inhibition; at 640-fold, it was 96.40 ± 4.50, indicating approximately 4% inhibition; at 320-fold, it was 63.50 ± 3.00, indicating approximately 36% inhibition; at 160-fold, it was 28.30 ± 1.70, indicating approximately 72% inhibition; at 80-fold, it was 16.00 ± 1.60, indicating approximately 84% inhibition; at 40-fold, it was 1.40 ± 0.20, indicating approximately 99% inhibition; and at 20-fold, it was 2.20 ± 0.20, indicating approximately 98% inhibition.

### 2.5. Viability of TGLON in MCF-7 Breast Cancer Cells

Refer to Figure 1D, where the viability of MCF-7 cells (% mean ± SD) was as follows: Blank (only cells) was 100.00 ± 5.50; at a 1280-fold dilution of TGLON, viability was 103.90 ± 5.30; at 640-fold, it was 113.00 ± 2.50; at 320-fold, it was 107.90 ± 6.60; at 160-fold, it was 107.30 ± 6.20; at 80-fold, it was 50.20 ± 3.30, indicating approximately 50% inhibition; at 40-fold, it was 2.30 ± 0.30, indicating approximately 98% inhibition; and at 20-fold, it was 3.60 ± 0.30, indicating approximately 96% inhibition.

### 2.6. Viability of TGLON in MKN-45 Stomach Cancer Cells

Refer to Figure 1E, where the viability of MKN-45 cells (% mean ± SD) was as follows: Blank (only cells) was 100.00 ± 0.02; at a 1280-fold dilution of TGLON, viability was 100.00 ± 0.03; at 640-fold, it was 83.00 ± 4.60, indicating approximately 17% inhibition; at 320-fold, it was 79.00 ± 8.10, indicating approximately 21% inhibition; at 160-fold, it was 72.00 ± 4.90, indicating approximately 28% inhibition; at 80-fold, it was 62.00 ± 6.10, indicating approximately 38% inhibition; at 40-fold, it was 31.00 ± 4.30, indicating approximately 69% inhibition; and at 20-fold, it was 22.00 ± 7.80, indicating approximately 78% inhibition.

### 2.7. Viability of TGLON in MOLT-4 Acute Lymphoblastic Leukemia Cells

Refer to Figure 1F, the viability of MOLT-4 cells (% mean ± SD) was as follows: Blank (only cells) was 100 ± 0.02; at a 2 × 10^5^-fold dilution of TGLON, viability was 99.80 ± 4.50; at 8 × 10^4^-fold, it was 92.90 ± 6.30, indicating approximately 7% inhibition; at 4 × 10^4^-fold, it was 85.00 ± 8.90, indicating approximately 5% inhibition; at 2 × 10^4^-fold, it was 72.00 ± 8.30, indicating approximately 28% inhibition; at 2 × 10^3^-fold, it was 64.00 ± 12.50, indicating approximately 36% inhibition; at 200-fold, it was 48.00 ± 1.20, indicating approximately 52% inhibition; and at 20-fold, it was 34.00 ± 1.40, indicating approximately 66% inhibition.

### 2.8. Acute Toxicity Assay of TGLON in Rats

Refer to Table 2, which shows that, during the two-week oral administration test of a 50% (*v*/*v*) TGLON solution at 10 mL/kg, no rat mortality or adverse clinical signs were recorded, nor were any gross necropsy lesions observed.

Refer to Table 3, which shows that, on Day 1, the body weight of male rats in Group 1 (WFI) was 287.5 ± 13.8 g, and in Group 2 (TGLON), it was 286.9 ± 14.1 g, demonstrating consistency in experimental conditions. On Day 15, the body weight of Group 1 (WFI) was 341.3 ± 24.3 g, with a final weight gain of 53.8 ± 16.0 g; the body weight of Group 2 (TGLON) on Day 15 was 336.5 ± 26.6 g, with a final weight gain of 49.6 ± 18.6 g.

The body weight of female rats on Day 1 in Group 1 (WFI) was 208.6 ± 10.0 g, and in Group 2 (TGLON), it was 205.6 ± 10.0 g, indicating consistent experimental conditions. On Day 15, the body weight of Group 1 (WFI) was 239.6 ± 8.1 g, with a final weight gain of 31.0 ± 8.6 g; the body weight of Group 2 (TGLON) on Day 15 was 238.6 ± 17.8 g, with a final weight gain of 33.0 ± 11.1 g. The results indicate that continuous oral administration of TGLON 50% (*v*/*v*) 10 mL/kg does not cause a decrease in rat body weight.

### 2.9. Investigation of TGLON in Treating IPF in Mice

**Figure 2:**
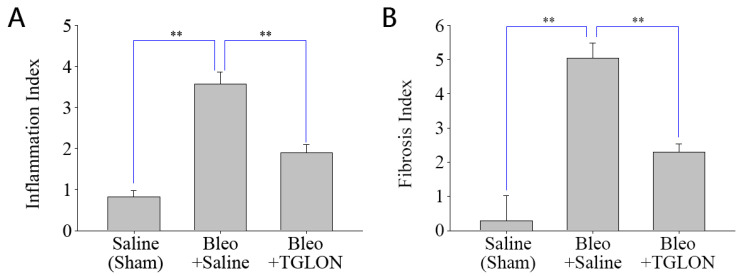
Inflammation index (**A**) and fibrosis index (**B**) in the lungs of mice treated with bleomycin. Data are presented as means ± SE. ** *p* < 0.01 compared to the Bleo+saline treated group. Bleo: bleomycin.

**Figure 3:**
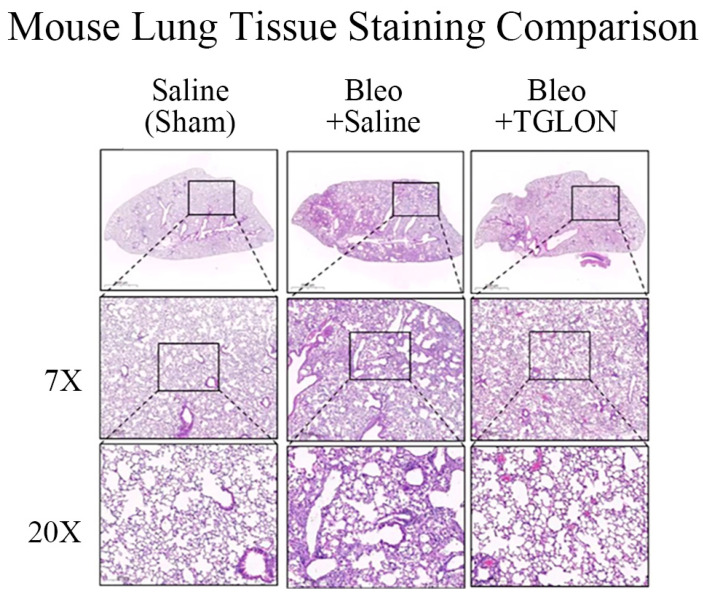
H&E staining of lung tissue in mice treated with bleomycin. ‘X’ refers to the specific magnification used when observing lung tissue. Due to the historical nature of the image acquisition, original scale bars were not retrievable. However, all images were captured under consistent magnification (7X and 20X), and the relative tissue morphology remains comparable. Fibrotic regions have been annotated to aid interpretation.

Refer to Figure 2A,B, which show that, in mice not induced with IPF and orally administered saline, the pulmonary inflammation index and fibrosis index were 0.82 ± 0.15 and 0.29 ± 0.74, respectively, indicating normal pulmonary tissue structure (Figure 3: Saline (Sham)). In contrast, mice induced with IPF using bleomycin and orally administered saline at 10 mL/kg for 21 days exhibited pulmonary inflammation and fibrosis indices of 3.57 ± 0.29 and 5.03 ± 0.45, respectively, indicating significant fibrosis and structural damage to the lung tissue, with the formation of fibrotic bands or small fibrotic clusters (Figure 3: Bleo + Saline). Conversely, when bleomycin-induced IPF mice were treated with a 50% (*v*/*v*) TGLON blend hydrosol at 10 mL/kg for 21 days, the pulmonary inflammation and fibrosis indices were 1.90 ± 0.19 and 2.30 ± 0.23, respectively. These values suggest moderate wall thickening but no significant structural damage, thereby mitigating the severity of bleomycin-induced IPF (Figure 3: Bleo + TGLON).

## 3. Discussion

In TGLON, the sum of relatively safe analogs such as sesquiterpenes, monoterpenes, monoterpenols, and sesquiterpenols accounts for 62.14%. This percentage is higher than the combined 22.79% of the more irritating ketones, aldehydes, and other oxides. Through the grouping of similar compounds, it has been established that TGLON meets our expectations for safety and mildness. The reason for classifying components by their analogs is as follows: the monoterpenol group exhibits anti-inflammatory properties; the ester group promotes the synthesis of elastin, exhibiting anti-wrinkle activity; and the monoterpene group inhibits HepG2 cancer cells. Therefore, similar structures tend to have similar activities, which is why we group components by analogs. This helps in systematically organizing and explaining the complex composition of the components [24,25,26,27,28,29].

In terms of cell safety, Terpinen-4-ol, Camphor, and δ-Cadinene in TGLON are key components in inhibiting cancer cells [30,31,32]. However, when the essential oil’s main component is Terpinen-4-ol, it causes 50% damage to normal lung cells under a 14,000-fold dilution [21]. Camphor causes 50% damage to normal lung cells at a 238,000-fold dilution [33]. δ-Cadinene causes 50% damage to normal lung cells at a 3900-fold dilution [34]. The literature indicates that even at such high dilutions, essential oils can harm normal lung cells, whereas TGLON blend hydrosol remains safe for normal lung cells even at higher concentrations (80-fold dilution). Interestingly, the safety of TGLON for MRC-5 cells may be due to the presence of Terpinen-4-ol, which inhibits the production of Tumor Necrosis Factor-Alpha (TNF-α), IL (Interleukin)-1β, IL-6, IL-10, and Prostaglandin E2 (PG-E2) induced by lipopolysaccharide (LPS) [25,35]; Camphor, which inhibits the expression of IL-1β, IL-6, and TNF-α [36,37]; and δ-Cadinene, which inhibits nitric oxide (NO) production induced by LPS [38]. Grouping by analogs helps us assess the potential to reduce risks to normal cells.

In terms of inhibiting cancer cells, TGLON has shown efficacy against A-549 cancer cells, potentially due to components such as Terpinen-4-ol, Camphor, δ-Cadinene, 1,8-Cineole, γ-Terpinene, and Cedrol. Notably, Cedrol can induce apoptosis in A-549 cancer cells by decreasing mitochondrial transmembrane potential (MTP) and inhibiting the expression of Phosphatidylinositol 3′-Kinase (PI3K)/Akt [39,40,41]. The inhibition of HepG2 cancer cells may be attributed to Terpinen-4-ol, γ-Terpinene, and α-Pinene in TGLON [42,43]. The inhibition of MCF-7 cancer cells may be due to Terpinen-4-ol, α-Terpineol, Camphor, and Safrole in TGLON [31,44,45,46]. The effects on MKN-45 cancer cells may be attributed to Camphor, 1,8-Cineole, and α-Pinene in TGLON [47]. The ability to inhibit MOLT-4 malignant cells may be due to Terpinen-4-ol and α-Pinene, whose primary mechanism involves the loss of mitochondrial membrane potential (MMP), cytochrome c release into the cytosol, and triggering of caspase-8 leading to cytosolic Bid cleavage, down-regulation of Bcl-2, and up-regulation of caspase-3 [48,49]. It should be emphasized that this study did not involve direct comparisons between TGLON and standardized compounds or clinically approved anticancer agents. Furthermore, due to limited resources, mechanistic studies—such as pathway analysis or molecular target validation—were not conducted. As a result, interpretations regarding potential synergistic or additive effects among the identified constituents remain hypothetical. This limitation is duly acknowledged, and future investigations are encouraged to address it through fractionation-based approaches and functional bioassays.

In acute toxicity assays, the 50% lethal dose (LD_50_) of Terpinen-4-ol administered orally to rats is 1.3 g/kg [50]; for Camphor, it is 1.31 g/kg [51]. When orally administered TGLON blend hydrosol 50% (*v*/*v*) at 10 mL/kg, it is well tolerated at higher doses without causing any deaths or poisoning, and body weight continues to grow normally, indicating higher safety compared to single compounds.

In vitro, TGLON maintained >90% viability in MRC-5 human lung fibroblasts at an 80-fold dilution. These findings indicate high biocompatibility at concentrations relevant to topical or oral application. Furthermore, in vivo oral administration at a 2-fold dilution (10 mL/kg) in mice resulted in no observable toxicity, thereby confirming the safety of TGLON under both cellular and systemic conditions.

TGLON’s ability to mitigate IPF can be attributed to the monoterpenol group components in TGLON such as α-Terpineol, (−)-Myrtenol, Borneol, cis-Myrtanol, and Terpinen-4-ol, as well as the aldehyde group component β-Citronellal. These components reduce malondialdehyde (MDA), alpha-smooth muscle actin (α-SMA), transforming growth factor-beta (TGF-β), cyclooxygenase-2 (COX-2), PG-E2, and TNF-α expressions while promoting the expression of SOD [4]. TGLON also shows similar effects to U.S.-approved drugs Nintedanib and Pirfenidone [52,53], providing a scientific basis for mitigating or preventing IPF indirectly caused by chronic lung diseases such as lung cancer.

## 4. Materials and Methods

### 4.1. Preparation of Blend Hydrosol and Chemicals

All plants were cultivated in the forest and farmland at altitudes ranging from 500 to 1000 m in Nantou County, Taiwan. The wood material, specifically CF, was processed through sawing and chipping, with 100 kg collected. For CM, CN, LC, CFS, CFA, CC, ERS, CZ, CL, MA, CMM, CJ, and AC, which comprise flowers, fruits, branches, leaves, and buds, 100 kg were similarly harvested. Subsequently, all plant materials underwent steam distillation as a single extraction process to obtain essential oils and hydrosols. During distillation, 100 kg of plant material yielded 100 L of hydrosol.

The formulation principle was based on major components such as Terpinen-4-ol, Camphor, and δ-Cadinene, known for their cancer cell inhibitory activities. In consideration of preventive medicine’s need for safer or milder approaches, the primary formulation was chosen to consist mainly of milder analogs like monoterpenes, sesquiterpenes, and sesquiterpenols, while reducing the proportion of more irritant analogs such as ketones, aldehydes, and other oxides. This composition created the blend hydrosol referenced in studies [30,31,32]. After experiencing the processes of cultivation, harvesting, and research, this blend hydrosol was named The Greatest Love of Nature (TGLON). Ultimately, the formulation of TGLON was CN 8%, LC 6%, CFS 40%, CFA 10%, CC 8%, ERS 6%, CZ 2%, CL 4%, MA 2%, CMM 10%, CJ 2%, and AC 2%.

Human MRC-5 lung cells (BCRC, 60023), A-549 lung cancer cells (BCRC, 60074), HepG2 liver cancer cells (BCRC, RM60025), MCF-7 breast cancer cells (BCRC, 60436), MKN-45 stomach cancer cells (ATCC, CRL1739), and MOLT-4 leukemic lymphoblasts (BCRC, 60060) were procured from the Bioresource Collection and Research Center (BCRC) in Hsinchu, Taiwan. All other chemicals used were of analytical reagent grade, sourced from ECHO CHEMICAL CO., LTD., Taichung City, Taiwan, and ultra-pure water was utilized throughout the analytical processes.

### 4.2. Gas Chromatography–Mass Spectrometry (GC-MS)

The methodology was adapted according to Adams (2017) [54]. The GC-MS analysis was conducted using a model 7890 gas chromatograph coupled with a 5977B mass spectrometer equipped with a quadrupole analyzer (Agilent Technologies, Inc., Santa Clara, CA, USA). Both instruments were operated and controlled using Agilent MassHunter Workstation software (version 10.1; Agilent Technologies Inc., Santa Clara, CA, USA), which enabled system operation, data acquisition, and compound identification. Chromatographic separation was performed on a DB-5 MS capillary column, 30 m in length, with an internal diameter of 0.25 mm and a film thickness of 0.25 µm (Agilent Technologies, Inc., USA). The injector was operated in split mode at a fixed temperature of 300 °C. The carrier gas was helium, 99.999% pure, with a flow rate set at 1 mL/min. The temperature program started at 50 °C and ramped to 280 °C at a rate of 3 °C per minute, followed by a 5 min isothermal hold. Mass spectrometric analysis was carried out using electron-impact ionization (EI) set at 70 eV and full-scan mode, covering a mass range from 40 to 450 *m*/*z*. The resulting mass spectra were analyzed using the National Institute of Standards and Technology (NIST) mass spectral database. Retention times were calculated using an *n*-alkane standard solution ranging from C_8_ to C_20_, and Kovats indices (KI) were compared with values reported in the literature [55]. Compound identification was further confirmed by calculating KI values using the following equation: $KI\;=\;100Z\;+\;100(\frac{{logt}^{\prime }R\left(x\right)-{logt}^{\prime }R\left(z\right)}{{logt}^{\prime }R\left(z+1\right)-{log}^{\prime }R\left(z\right)}).$

### 4.3. In Vitro Cytotoxicity Assay

The methodology was refined from the protocol described by Alle et al. (1988) [56]. Initially, preliminary microscopic inspection and reference to growth curves were used to calculate the initial seeding densities. Cells were then seeded in 96-well plates as follows: MRC-5, A-549, HepG2, and MCF-7 cells at 3000 cells per well; MOLT-4 cells at 10,000 cells per well; and MKN-45 cells at 12,500 cells per well. Each well received 100 μL of culture medium and was incubated for 24 h. Subsequently, the culture medium was aspirated, and test samples at various concentrations were added to the wells. Dosages were set for MRC-5, A-549, HepG2, MCF-7, and MKN-45 cells as follows: Blank (cells only), 20-fold, 40-fold, 80-fold, 160-fold, 320-fold, 640-fold, and 1280-fold. And, for MOLT-4 cells, dosages were set as follows: Blank (cells only), 20-fold, 200-fold, 2 × 10^3^-fold, 2 × 10^4^-fold, 4 × 10^4^-fold, 8 × 10^4^-fold, and 2 × 10^5^-fold. Cultivation continued for 72 h before the culture medium was again aspirated and the wells were washed with 150 μL/well of phosphate-buffered saline (PBS). Subsequently, 100 μL of MTT reagent (0.5 mg/mL) was added to each well, covered with aluminum foil to protect from light, and the plates were placed in a CO_2_ incubator for 30 min. After removal, the MTT reagent was aspirated and the wells were rinsed with PBS. Finally, 100 μL/well of DMSO was added to dissolve the formazan crystals, and the absorbance was measured at 540 nm using an ELISA reader. Cells were maintained in media appropriate for their growth characteristics: MRC-5 in Minimum Essential Medium (MEM); A-549, HepG2, and MCF-7 in Dulbecco’s Modified Eagle Medium (DMEM); and MOLT-4 and MKN-45 in Roswell Park Memorial Institute 1640 Medium (RPMI-1640). All media were supplemented with 10% Fetal Bovine Serum (FBS).

### 4.4. In Vivo Acute Oral Toxicity Study

In this study, Crl: CD Sprague Dawley (SD) rats from BioLASCO Taiwan Co., Ltd., Ilan, Taiwan, were used as model organisms. Selection criteria included age (8 to 9 weeks), with male rats weighing between 260 and 300 g and female rats weighing between 180 and 230 g to ensure consistency in the study. The choice of SD rats was based on their favorable response to various toxicological agents, making them ideal for toxicity assessments. Ethical considerations were paramount, and the animal experiments were rigorously evaluated and approved by the Institutional Animal Care and Use Committee (IACUC), with the approval number 2020-R501-051, on 23 December 2020. We strictly adhered to globally recognized standards for the care and use of laboratory animals to ensure ethical and responsible scientific conduct. Prior to the start of the study, all animals underwent a minimum three-day quarantine and acclimation period in the animal facility.

As for the housing conditions, three rats were kept in each polycarbonate cage, with details such as group number, study number, dosage level, gender, animal ID, and planned euthanasia date labeled. Environmental conditions were maintained at a room temperature of 22 ± 3 °C, with a relative humidity of 50 ± 20%, and a 12 h light/dark cycle, with lights on at 7:00 and off at 19:00. The rats were fed PicoLab^®^ Rodent Diet 20 (LabSupply Inc., Fort Worth, TX, USA) ad libitum throughout the study. Sterilized tap water was made available at all times in hanging cages for free consumption by the rats.

During the experiment, the rats were administered orally with a 50% (*v*/*v*) solution of TGLON at 10 mL/kg, or with water for injection (WFI) as a control. Body weight changes were monitored on Day 1, Day 8, and Day 15, along with recording mortality and clinical signs throughout the study. Finally, necropsies were conducted on Day 15 to observe any potential damage [57].

### 4.5. In Vivo Idiopathic Pulmonary Fibrosis (IPF) Study

In this investigation, 18 male C57BL/6 mice (BioLASCO Taiwan Co., Ltd., Ilan, Taiwan) were utilized as model organisms. Selection criteria were age (8 to 9 weeks) and weight range (20 to 25 g) to ensure consistency in the study. The animal experiments were rigorously evaluated and approved by the IACUC, with approval number 2021-R501-049, on 7 April 2021. Each mouse was identified by ear tags and cage numbers. Animals were housed in polycarbonate cages, with three mice per cage. Each cage was equipped with an identification card that recorded the cage number, trial number, dosage group, gender, and animal number. Environmental conditions were maintained at a room temperature of 22 ± 3 °C, relative humidity of 50 ± 20%, and a 12 h automatic light/dark cycle, with lights turning on at 7:00 and off at 19:00. The mice were fed PicoLab^®^ Rodent Diet 20 (LabSupply Inc., USA) ad libitum throughout the study. Sterilized tap water was provided in hanging cages for free consumption. Mice were randomly assigned to three groups: a healthy control group, a disease control group induced by bleomycin, and a treatment group receiving TGLON.

During the experiment, mice received oral administration of TGLON at 50% (*v*/*v*) or saline (1 mL/kg) once daily for 2 weeks. Subsequently, idiopathic pulmonary fibrosis (IPF) was induced via intratracheal instillation of bleomycin (50 μL, 3 U/kg). Oral administration of TGLON or saline continued for an additional 3 weeks. At the end of the treatment period, mice were euthanized, and the left lung lobe was harvested from each animal. Tissues were fixed in 4% paraformaldehyde for 24 h, embedded in paraffin, and transversely sectioned at a thickness of 4–6 μm. The sections were stained with hematoxylin and eosin (H&E) for histopathological evaluation of inflammation and tissue architecture [58,59].

### 4.6. Statistical Analysis

Calculations of means, standard errors (SE), and standard deviations (SD) were performed using Microsoft Excel. Statistical analyses were conducted using the Statistical Package for the Social Sciences (SPSS), version 16. One-way analysis of variance (ANOVA) followed by Duncan’s multiple range test was applied to evaluate differences in cell viability among various dilution groups. For in vivo IPF experiments involving two groups, the Student’s *t*-test was used to assess significance. Differences were considered statistically significant at *p* < 0.05 and highly significant at *p* < 0.01.

## 5. Conclusions

The safety profile of the blend hydrosol TGLON was initially evaluated using MRC-5 normal human lung fibroblasts, with cell viability exceeding 90% at an 80-fold dilution, thereby confirming a non-cytotoxic concentration. At this dilution, TGLON significantly inhibited the proliferation of several human cancer cell lines, including lung (A-549), liver (HepG2), breast (MCF-7), gastric (MKN-45), and acute lymphoblastic leukemia (MOLT-4) cells. In vivo, oral administration of TGLON at 50% (*v*/*v*), 1 mL/kg, showed no signs of acute toxicity and effectively attenuated idiopathic pulmonary fibrosis (IPF) in mice.

These findings support the potential of TGLON as a safe and bioactive candidate for development in preventive medicine, particularly for non-clinical health maintenance and early-stage disease intervention. However, further investigations are required to elucidate the complete chemical profile of the hydrosol, including non-volatile constituents not detected by GC-MS analysis, and to evaluate its long-term physicochemical stability. Moreover, the inclusion of appropriate reference standards and positive controls in future studies will be essential for efficacy benchmarking and regulatory advancement.

## 6. Patents

The authors would like to express their gratitude to the intellectual property offices of China, Taiwan, and the United States for their recognition of this study and the approval of patent numbers CN106668571B, TWI605818B, and US10314872B2.

## Figures and Tables

**Table 1:** GC-MS analysis of TGLON.

No.	R. Time	Constituent	Analog Type	Mol. Form	% Area
1	9.606	α-Pinene	monoterpene	C_10_H_16_	2.46
2	12.221	α-Terpinene	monoterpene	C_10_H_16_	1.54
3	12.455	para-Cymene	monoterpene	C_10_H_14_	1.42
4	12.603	D-Limonene	monoterpene	C_10_H_16_	1.93
5	12.669	1,8-Cineole	aother oxide	C_10_H_18_O	6.08
6	13.552	γ-Terpinene	monoterpene	C_10_H_16_	3.17
7	16.174	Camphor	ketone	C_10_H_16_O	8.73
8	16.412	β-Citronellal	aldehyde	C_10_H_18_O	1.11
9	16.83	Borneol	monoterpenol	C_10_H_18_O	1.12
10	17.177	Terpinen-4-ol	monoterpenol	C_10_H_18_O	10.72
11	17.555	α-Terpineol	monoterpenol	C_10_H_18_O	6.93
12	17.734	(−)-Myrtenol	monoterpenol	C_10_H_16_O	3.94
13	19.559	cis-Myrtanol	monoterpenol	C_10_H_18_O	4.22
14	20.361	Safrole	aother oxide	C_10_H_10_O_2_	6.87
15	23.258	β-Elemene	sesquiterpene	C_15_H_24_	0.94
16	23.86	α-Cedrene	sesquiterpene	C_15_H_24_	2.47
17	26.013	γ-Muurolene	sesquiterpene	C_15_H_24_	2.15
18	26.373	α-Muurolene	sesquiterpene	C_15_H_24_	2.9
19	26.569	δ-Cadinene	sesquiterpene	C_15_H_24_	7.58
20	27.173	α-Elemol	sesquiterpenol	C_15_H_26_O	1.45
21	28.519	α-Cedrol	sesquiterpenol	C_15_H_26_O	2.16
22	29.365	tau-Cadinol	sesquiterpenol	C_15_H_26_O	2.82
23	29.663	tau-Muurolol	sesquiterpenol	C_15_H_26_O	2.46
			Sesquiterpenes		16.04
			Monoterpenes		10.52
			Monoterpenols		26.93
			Sesquiterpenols		8.89
			Ketones		8.73
			Aldehydes		1.11
			Other oxides		12.95

**Table 2:** Mortality, clinical observations, and gross findings in rats.

Gender	Male		Female	
Group	1 ^a^	2 ^b^	1 ^a^	2 ^b^
Mortality	0/5	0/5	0/5	0/5
Treatment-related Clinical Signs	0/5	0/5	0/5	0/5
Treatment-related Gross Necropsy Lesion	0/5	0/5	0/5	0/5

^a^: water for injection (WFI) 10 mL/kg. ^b^: TGLON 50% (*v*/*v*) 10 mL/kg.

**Table 3:** Body weight variations in rats.

	Body Weights			
Gender	Male		Female	
Group	1 ^a^	2 ^b^	1 ^a^	2 ^b^
Day 1	287.5 ± 13.8	286.9 ± 14.1	208.6 ± 10.0	205.6 ± 10.0
Day 8	317.6 ± 19.5	320.2 ± 21.7	225.6 ± 10.0	227.9 ± 16.3
Day 15	341.3 ± 24.3	336.5 ± 26.6	239.6 ± 8.1	238.6 ± 17.8
Final Gain	53.8 ± 16.0	49.6 ± 18.6	31.0 ± 8.6	33.0 ± 11.1

Final gain: body weight on Day 15 minus body weight on Day 1 (Mean ± SD, *N* = 5). ^a^: water for injection (WFI) 10 mL/kg. ^b^: TGLON 50% (*v*/*v*) 10 mL/kg.

## Data Availability

The data that support the findings of this study are openly available in Mendeley Data at https://data.mendeley.com/datasets/kmvxghygkv/1 (accessed on 16 April 2024).

## Supplementary Materials

The following supporting information can be downloaded at: https://www.mdpi.com/article/10.3390/ph18060872/s1. Figure S1. Fingerprint chromatogram of TGLON. Table S1. Major phytochemical constituents of the TGLON.

## Abbreviations

The following abbreviations are used in this manuscript:

AC	*Acacia confusa*
ANOVA	Analysis of Variance
ASR	Age-standardized Incidence Rate
BCRC	Bioresource Collection and Research Center
CC	*Cinnamomum camphora*
CDSD	CD Sprague Dawley
CFA	*Calocedrus formosana*
CFS	*Chamaecyparis formosensis*
CJ	*Cryptomeria japonica*
CL	*Cunninghamia lanceolata*
CM	*Cinnamomum micranthum*
CN	*Cymbopogon nardus*
COX-2	Cyclooxygenase-2
CZ	*Cinnamomum zeylanicum*
DMEM	Dulbecco’s Modified Eagle Medium
ECM	Extracellular Matrix
EI	Electron-impact Ionization
ERS	*Eucalyptus robusta*
FBS	Fetal Bovine Serum
GC-MS	Gas Chromatography–Mass Spectrometry
H&E	Hematoxylin and Eosin
IACUC	Institutional Animal Care and Use Committee
IL	Interleukin
IPF	Idiopathic Pulmonary Fibrosis
KI	Kovats indices
LC	*Litsea cubeba*
LD_50_	Lethal Dose 50%
LPS	Lipopolysaccharide
MA	*Melaleuca alternifolia*
MDA	Malondialdehyde
MEM	Minimum Essential Medium
MMP	Mitochondrial Membrane Potential
MTP	Mitochondrial Transmembrane Potential
NIST	National Institute of Standards and Technology
NO	Nitric Oxide
PBS	Phosphate-buffered saline
PG-E2	Prostaglandin E2
PI3K	Phosphatidylinositol 3′-Kinase
RPMI-1640	Roswell Park Memorial Institute 1640 medium
SD	Standard Deviation
SE	Standard Error
SPSS	Statistical Package for the Social Sciences
TGF-β	Transforming Growth Factor-Beta
TGLON	The Greatest Love of Nature
TNF-α	Tumor Necrosis Factor-Alpha
WFI	Water for injection
α-SMA	Alpha-smooth Muscle Actin
