# Supplementary material for: Potential Natural Blend Hydrosol TGLON Suppresses the Proliferation of Five Cancer Cell Lines and Also Ameliorates Idiopathic Pulmonary Fibrosis in a Mouse Model
Source: Pharmaceuticals (Basel). 2025 Jun 11;18(6):872. doi: 10.3390/ph18060872 (PMC12196324; doi:10.3390/ph18060872)
Supplement: Supplementary file 1 [file pharmaceuticals-18-00872-s001.zip › pharmaceuticals-3625083-supplementary.pdf]

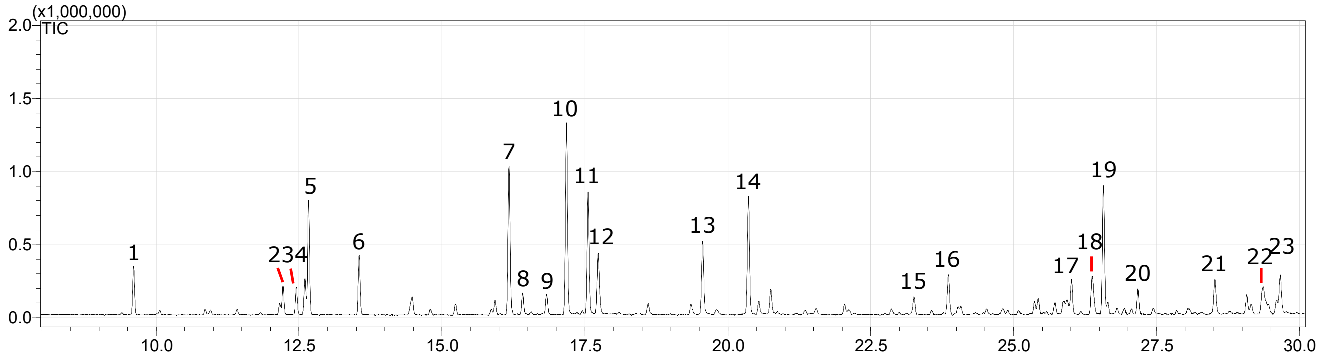

Figure S1. Fingerprint chromatogram of TGLON.

Table S1. Major phytochemical constituents of the TGLON.

No.	Structureof Compound	Common Name	KI	R. time	% Area	Method of Identification
1	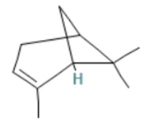	α-Pinene	939	9.606	2.46	MS;KI;RC
2	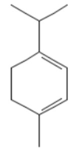	α-Terpinene	1017	12.221	1.54	MS;KI
3	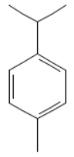	para-Cymene	1024	12.455	1.42	MS;KI;RC
4	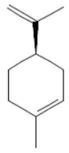	D-Limonene	1029	12.603	1.93	MS;KI;RC
5	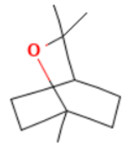	1,8-Cineole	1031	12.669	6.08	MS;KI;RC
6	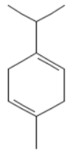	γ-Terpinene	1059	13.552	3.17	MS;KI

7	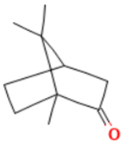	Camphor	1146	16.174	8.73	MS;KI;RC
8	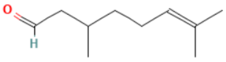	β-Citronellal	1153	16.412	1.11	MS;KI
9	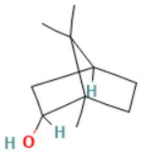	Borneol	1169	16.83	1.12	MS;KI;RC
10	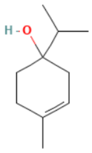	Terpinen-4-ol	1177	17.177	10.72	MS;KI;RC
11	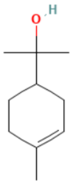	α-Terpineol	1188	17.555	6.93	MS;KI;RC
12	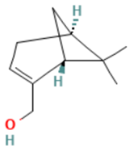	(-)-Myrtenol	1195	17.734	3.94	MS;KI
13	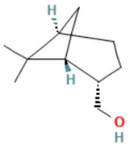	cis-Myrtanol	1253	19.559	4.22	MS;KI
14	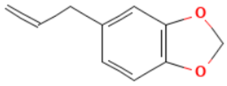	Safrole	1287	20.361	6.87	MS;KI
15	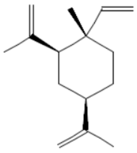	β-Elemene	1390	23.258	0.94	MS;KI
16	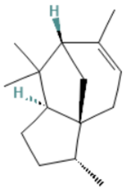	α-Cedrene	1411	23.86	2.47	MS;KI

17	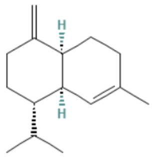	γ-Muurolene	1479	26.013	2.15	MS;KI
18	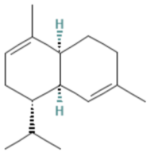	α-Muurolene	1500	26.373	2.9	MS;KI
19	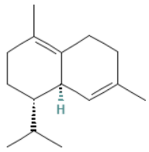	δ-Cadinene	1523	26.569	7.58	MS;KI
20	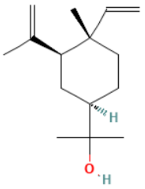	α-Elemol	1549	27.173	1.45	MS;KI
21	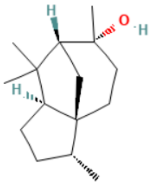	α-Cedrol	1601	28.519	2.16	MS;KI;RC
22	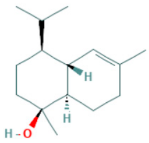	tau-Cadinol	1640	29.365	2.82	MS;KI
23	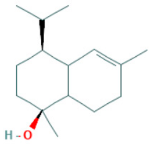	tau-Muurolol	1642	29.663	2.46	MS;KI
